# Complications Risk Assessment and Imaging Findings of Thermal Ablation Treatment in Liver Cancers: What the Radiologist Should Expect

**DOI:** 10.3390/jcm11102766

**Published:** 2022-05-13

**Authors:** Vincenza Granata, Roberta Fusco, Federica De Muzio, Carmen Cutolo, Sergio Venanzio Setola, Igino Simonetti, Federica Dell’Aversana, Francesca Grassi, Federico Bruno, Andrea Belli, Renato Patrone, Vincenzo Pilone, Antonella Petrillo, Francesco Izzo

**Affiliations:** 1 Radiology Division, Istituto Nazionale Tumori—IRCCS—Fondazione G. Pascale, Via Mariano Semmola, 80131 Naples, Italy; s.setola@istitutotumori.na.it (S.V.S.); igino.simonetti@istitutotumori.na.it (I.S.); a.petrillo@istitutotumori.na.it (A.P.); 2 Medical Oncology Division, Igea SpA, 80013 Naples, Italy; r.fusco@igeamedical.com; 3 Department of Medicine and Health Sciences V. Tiberio, University of Molise, 86100 Campobasso, Italy; demuziofederica@gmail.com; 4 Department of Medicine, Surgery and Dentistry, University of Salerno, 84084 Fisciano, Italy; carmencutolo@hotmail.it (C.C.); vpilone@unisa.it (V.P.); 5 Division of Radiology, Università degli Studi della Campania Luigi Vanvitelli, 80138 Naples, Italy; federica.dellaversana@studenti.unicampania.it (F.D.); francescagrassi1996@gmail.com (F.G.); 6 Italian Society of Medical and Interventional Radiology (SIRM), SIRM Foundation, 20122 Milan, Italy; federico.bruno.1988@gmail.com; 7 Department of Applied Clinical Sciences and Biotechnology, University of L’Aquila, 67100 L’Aquila, Italy; 8 Hepatobiliary Surgical Oncology Division, Istituto Nazionale Tumori—IRCCS—Fondazione G. Pascale, Via Mariano Semmola, 80131 Naples, Italy; a.belli@istitutotumori.na.it (A.B.); dott.patrone@gmail.com (R.P.); f.izzo@istitutotumori.na.it (F.I.)

**Keywords:** RFA, MWA, HCC, liver metastases, complications, imaging

## Abstract

One of the major fields of application of ablation treatment is liver tumors. With respect to HCC, ablation treatments are considered as upfront treatments in patients with early-stage disease, while in colorectal liver metastases (CLM), they can be employed as an upfront treatment or in association with surgical resection. The main prognostic feature of ablation is the tumor size, since the goal of the treatment is the necrosis of all viable tumor tissue with an adequate tumor-free margin. Radiofrequency ablation (RFA) and microwave ablation (MWA) are the most employed ablation techniques. Ablation therapies in HCC and liver metastases have presented a challenge to radiologists, who need to assess response to determine complication-related treatment. Complications, defined as any unexpected variation from a procedural course, and adverse events, defined as any actual or potential injury related to the treatment, could occur either during the procedure or afterwards. To date, RFA and MWA have shown no statistically significant differences in mortality rates or major or minor complications. To reduce the rate of major complications, patient selection and risk assessment are essential. To determine the right cost-benefit ratio for the ablation method to be used, it is necessary to identify patients at high risk of infections, coagulation disorders and previous abdominal surgery interventions. Based on risk assessment, during the procedure as part of surveillance, the radiologists should pay attention to several complications, such as vascular, biliary, mechanical and infectious. Multiphase CT is an imaging tool chosen in emergency settings. The radiologist should report technical success, treatment efficacy, and complications. The complications should be assessed according to well-defined classification systems, and these complications should be categorized consistently according to severity and time of occurrence.

## 1. Introduction

The management of oncological patients has changed profoundly and, although the main goal is overall survival (OS), new knowledge of the molecular cancer profile and the development of new pharmacologic treatments has led to the use of surrogate end-points to measure interim treatment efficacy [1,2,3,4,5,6,7] related to the disease setting, including disease-free (DF), recurrence-free (RF), and progression-free survival (PFS) [8,9,10,11,12]. In this context, interventional radiology (IR), especially ablation treatment, is one of the most rapidly growing areas in oncology [13,14,15,16]. Its success is essentially due to the minimally invasive nature of the treatment with lower complication rates and superior toxicity profiles, and often with comparable or superior mid- and long-term oncologic outcomes compared to conventional therapies, such as surgical procedures or systemic treatments [17,18,19,20].

One of the major fields of application of ablation treatment is liver tumors [21,22,23,24], with hepatocellular carcinoma (HCC) and metastases representing the principal targets [25,26,27,28,29,30,31]. With respect to HCC, ablation treatments are considered as upfront treatments in patients with early-stage disease [24]. Today, ablation treatments are utilized in colorectal liver metastases (CLM) [32,33,34,35] as an upfront treatment or in association to surgical resection [24]. With respect to HCC, the feasibility of treatment is correlated to the number of nodules; in a metastatic setting, the number of lesions does not impact on the treatment [24].

The main prognostic feature of ablation is the tumor size, since the goal of this treatment is the necrosis of all viable tumor tissue with an adequate tumor-free margin [24,36,37,38,39,40,41]. Considering currently available devices, the target area should not exceed 3–4 cm for the longest diameter [36,37].

Radiofrequency ablation (RFA) and microwave ablation (MWA) are the most employed ablation techniques, whereas electroporation-based treatments, i.e., electrochemotherapy (ECT) and irreversible electroporation (IRE), have recently emerged as possible alternatives, due to their non-thermal nature [42,43,44,45,46,47,48,49,50,51].

RFA and MWA are hyperthermic procedures, heating tissues to at least 60 °C for maximum efficacy [52]. Although, the technical features of these percutaneous treatments are similar, they differ physically on the basis of heat generation [24]. RFA causes cell death by thermocoagulation while MWA uses dielectric heating. For MWA heat is contained in a volume around the applicator antenna, while for RFA it is limited to areas of high current density (Table 1) [24].

Ablation therapies have created a challenge for radiologists, who need to assess response to assess complications related to treatment [52,53,54,55,56,57,58,59,60,61].

The aim of this paper is to report the main challenges for radiologists in the assessment of ablation treatment complications, including the standardization of radiological reports.

## 2. Imaging and Ablation Treatment

During ablation treatments, imaging is used at five separate and distinct stages: planning, staging, monitoring, intra-procedural modification and assessment of treatment response, including technical success, treatment efficacy and complications [62,63,64,65,66,67,68,69,70,71,72,73].

The term “technical success” refers to the possibility of treating the target according to the protocol, covering the entire lesion in order to help searches to separate those patients in whom the protocol could not be fully performed from those who were treated according to the protocol [61].

The term “technique efficacy”, that may be differentiated from “technical success”, refers to the “complete ablation” of a macroscopic lesion and can be demonstrated with appropriate imaging follow-up at a defined time point [61].

Complications, identified as any unexpected variation from a procedural course, and adverse events, identified as any actual or potential injury related to the treatment, should be assessed according to the following classification systems: (a) the Common Terminology Criteria for Adverse Events standards, (b) the Clavien–Dindo classification, (c) the Society of Interventional Radiology classification, and (d) the Cardiovascular and Interventional Radiological Society of Europe Quality Assurance Document and Standards for Classification of Complications [59], and these complications should be characterized according to the gravity and the occurrence time (e.g., during treatment, post-treatment, or late) [60,61].

Different diagnostic tools may be employed, alone or in association [62,63,64,65,66,67,68,69,70,71,72,73]. Computed tomography (CT) and magnetic resonance imaging (MRI) represent the traditional imaging tools employed during the pre-treatment phase in order to identify and assess the target area [74,75,76,77,78,79,80,81,82,83,84,85,86], and in surveillance of the patient to evaluate treatment efficacy and post-procedural complications [87,88,89,90,91,92,93]. Ultrasound examination (US), without or with contrast medium (CEUS), is an innovative tool, utilized for problem solving during pre- and post-treatment phases [94,95,96,97,98,99,100,101,102,103,104], although the main use of interest is the possibility of assessing immediate effects during the procedure [60,61]. Given the ability of CEUS to detect real-time perfusion during the treatment, and considering the advantages of higher temporal resolution and the possibility of repeating an examination several times in a short period, it represents a secure and cost-effective tool for procedure outcome evaluation [96].

## 3. Complications and Risk Assessment

Complications, defined as any unexpected variation from a procedural course, and adverse events, defined as any actual or potential injury related to the treatment, could occur during the procedure or afterwards [24].

A major complication is an event that leads to substantial morbidity and disability, increasing the level of care or resulting in hospital admission or a substantially lengthened hospital stay. Events different from this scenario are minor complications [24].

According to Izzo et al. [24], with regard to HCC, OS, liver recurrence, complication rates, DFS and mortality in patients treated with MWA (with respect to RFA) varied between 22 months for lesions >3 cm (vs. 21 months) and 50 months for lesions ≤3 cm (vs. 27 months), between 5% (vs. 46.6%) and 17.8% (vs. 18.2%), between 2.2% (vs. 0%) and 61.5% (vs. 45.4%), between 14 months (vs. 10.5 months) and 22 months (vs. no data reported), and between 0% (vs. 0%) and 15% (vs. 36%), respectively [24]. With regard to liver metastases, for OS, there was no statistically different between the techniques for survival times from primary tumor diagnosis and survival times from ablation; liver recurrence, complication rates, and mortality in patients treated with RFA (vs. MWA), varied between 10% (vs. 6%) and 35.7% (vs. 39.6), between 1.1% (vs. 3.1%) and 24% (vs. 27%), and between 0% (vs. 0%) and 2% (vs. 0.3%), respectively.

Since RFA and MWA are thermal procedures, they could cause thermal damage. It is crucial, therefore, that an accurate risk assessment, based on patients and lesion characteristics, is performed [25,60,61].

**Figure 1:**
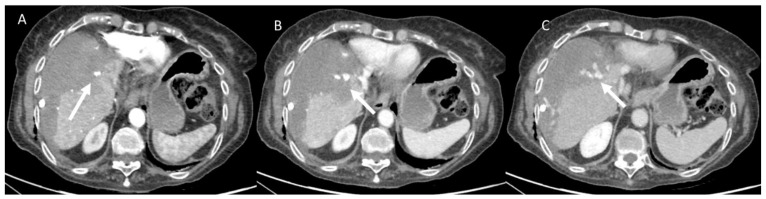
Woman 67 years at 1-day follow-up after radio frequency ablation of liver metastases. CT assessment (**A**) arterial phase; (**B**) portal phase and (**C**) late phase: active bleeding is present (arrow).

**Figure 2:**
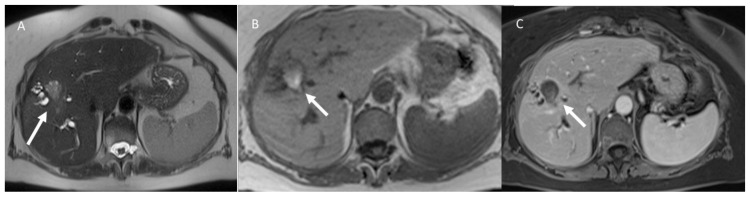
Woman 58 years at 1-month follow-up after microwave ablation of liver metastasis. MRI assessment (**A**) half-Fourier acquisition single-shot turbo-spin-echo (HASTE) T2-weighted sequence; in phase T1-weigthed sequence pre (**B**,**C**) post contrast assessment: ablated zone with biliary tree damage (arrow).

With regard to patients, both HCC patients and colorectal liver metastases patients [105,106,107,108,109,110,111,112,113] could have impaired liver function due to cirrhosis or drug-induced liver injury [114], so, they are at increased risk of bleeding (Figure 1) or biliary damage (Figure 2). In addition, in immunocompromised patients, the risk of infection of the ablated area is high, with consequent risk of liver abscess [115,116,117,118,119,120,121,122,123,124].

**Figure 3:**
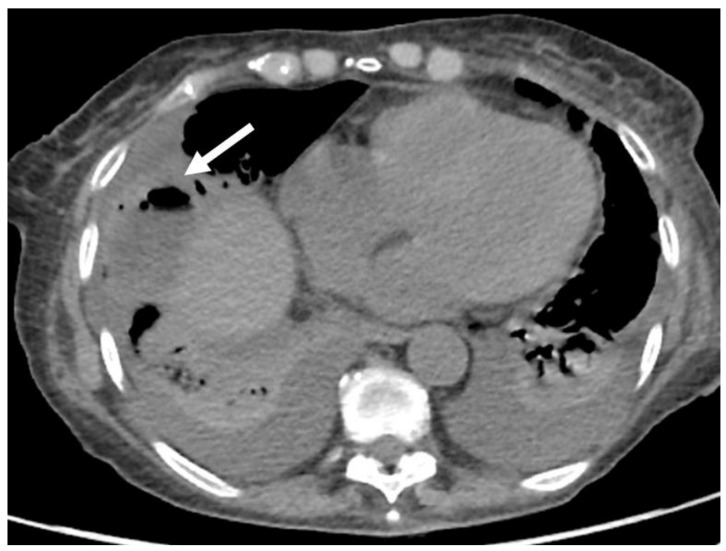
CT assessment at 1-week follow-up after radio frequency of HCC located on segment VIII. The arrow shows pulmonary abscess in patient with diaphragm damage.

**Figure 4:**
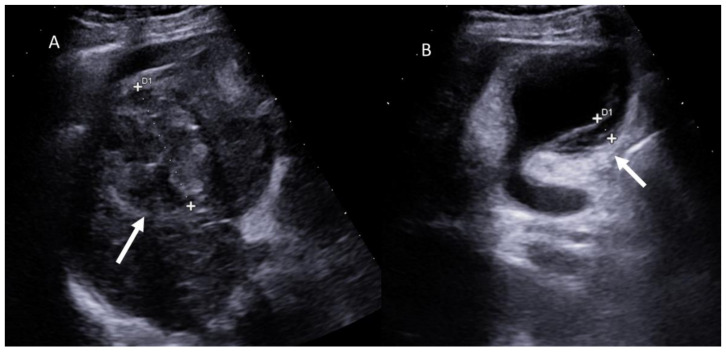
Ultrasound assessment of radio frequency treated HCC on V segment ((**A**) arrow). In (**B**) arrow shows cholecystitis.

With regard to lesion characteristics, according to Izzo et al. [24], lesions located in segment VIII are often the most challenging due to being near the diaphragm. It was reported that there was a 20% failure rate for lesions of segment VIII [125]. Abe et al. described complete ablation of HCC on VIII seg. in 9 of 15 lesions. In their cases, among the six lesions that had incomplete necrosis, two were near the diaphragm. The authors do not recommend MWA treatment for lesions that are in contact with the diaphragm because there was an increased risk of incomplete ablation, diaphragm injury (Figure 3), and pneumothorax [125]. Additionally, the researchers recommended MWA treatment for lesions that are in contact with the gallbladder, combining this procedure with laparoscopic cholecystectomy, due to gallbladder perforation or cholecystitis risk (Figure 4). Conversely, Simo et al. [126] showed that, in their case series, the treatment of two lesions that were in contact with the gallbladder, caused complete necrosis without complications.

Other significant issues include proximity of large blood and biliary vessels, proximity to extrahepatic structures, such as the pleura or/and intestine, as well as the lesion size. Schullian et al., using multivariable logistic regression analysis, showed that bile duct surgery/intervention history, number of coaxial needles, and tumor location in IVa or VIII segments, were independent prognostic features correlated with major complications. Logistic regression analysis showed that tumor number, size and shape (for tumor conglomerates), and location close to the diaphragm and segment VII were other significant predictors of complications [120].

To date, no statistically significant differences in mortality rates or major or minor complications for RFA and MWA have been shown. To reduce the rate of major complications, patient selection and risk assessment are essential. To establish the right cost-benefit ratio of the ablation method to be used, it is necessary to identify patients at high risk of infections, coagulation disorders and who have had previous abdominal surgery interventions [24]. Gastro-enteric perforation or biliary damage should be avoided by means of thermocouples to check the temperature and to ensure opportune consented treatment stoppage.

In this context, it is clear that an accurate patient assessment, based on clinical and laboratory data (e.g., re immunocompromised patients, impaired liver function, platelet count, etc.), as well as lesion assessment, including tumor location in segment IVa or VIII, contact with the gallbladder, proximity of large blood and biliary vessels, proximity to extrahepatic structures, such as the pleura or/and intestine, as well as the lesion size, is required in order to define proper patient management and to enable selection of alternative non-thermal treatment.

## 4. Radiologist: What Do We Expect?

Based on risk assessment, during surveillance of the procedure, the radiologist should pay attention to particular consequences, such as vascular, biliary, mechanical and infectious complications.

Vascular complications occur in 0.1–0.4% of cases [126,127,128,129], including hemorrhage, arteriovenous fistula, hepatic arterial pseudoaneurysm, portal vein thrombosis, and hepatic infarction [129]. Bleeding and pseudoaneurysm are the result of vessel injury by mechanical force from the needle or indirect thermal injury. Lesions that are closer to the main vessels and are subcapsular in location also have an increased risk of bleeding [129].

Pseudoaneurysm is found in follow-up imaging study as an incidental finding [126].

Hepatic or peritoneal hemorrhage represents the most frequent vascular complication, requiring an accurate imaging assessment. It is possible to evaluate this condition with CEUS, although in an emergency setting [130,131,132,133,134,135,136], a CT with multiphase contrast study is the diagnostic tool to choose [136,137,138,139].

Hemorrhage can involve the biliary tree, and, in the event of cystic duct obstruction by blood clots, it can cause cholecystitis [126].

During follow-up, it is possible to detect small sub-capsular or parenchymal haematomas, such as those arising from arterial-portal shunting due to needle injury. Arterial-portal shunting in patients with chronic disease could cause a flow dynamics modification with consequent decompensation. At CT assessment, it is possible to detect ascites (a sign of decompensation) and early portal vein contrast enhancement during the arterial phase of the contrast study [126].

Partial or complete portal vein thrombosis has been reported when treating targets near to the main portal vein or its major branches. In complete portal vein thrombosis, mesentery ischemia could occur. Hepatic arterial and hepatic venous thrombosis have also been described [126].

**Figure 5:**
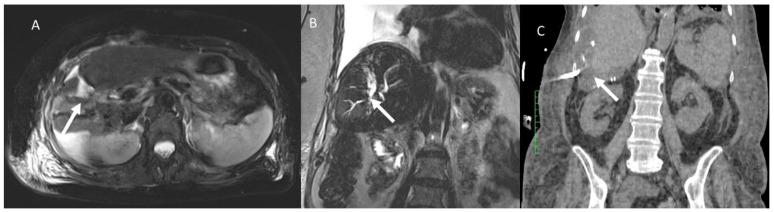
Woman 58 years at 1-week follow-up after microwave ablation of liver metastasis. MRI assessment. (**A**) Sampling perfection with application optimized contrasts using different flip angle evolution (SPACE) T2-weighted fat sat sequence in axial plane and (**B**) SPACE T2-weighted fat sat sequence in coronal plane, arrow shows biloma. In (**C**) CT evaluation (multi-planar reconstruction coronal plane) of biloma drainage (arrow).

**Figure 6:**
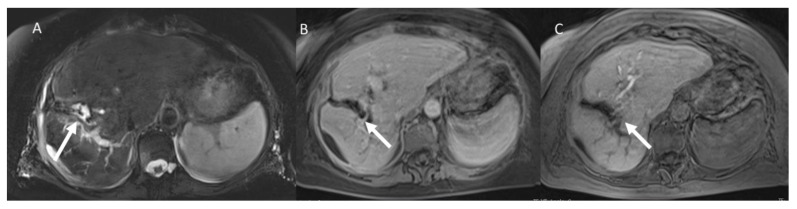
The same patient as Figure 5. MRI assessment. (**A**) SPACE T2-weighted sequence, (**B**): portal phase of contrast study and (**C**) EOB-phase after 1-month, arrow shows biloma and no bile leak.

**Figure 7:**
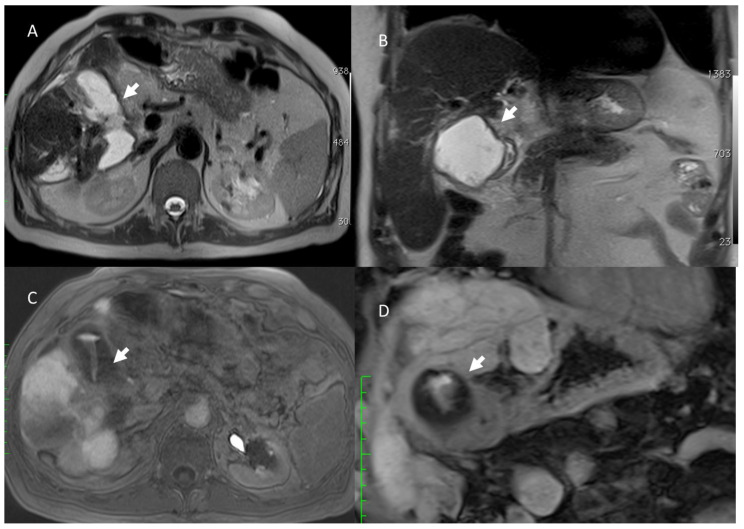
Man 74 years at 1-month follow-up after radio frequency of HCC on VI segment. MRI ((**A**,**B**) HASTE T2-weighted sequences in axial and coronal plane, in (**C**,**D**) EOB-phase of contrast study in axial and coronal plane). The arrow shows bile leak.

Biliary complications include biliary strictures, biloma (Figure 5 and Figure 6), bile leak (Figure 7) and acute cholecystitis.

Treatment of lesions in the central liver near large biliary ducts may predispose patients to clinically significant biliary stricture formation, while biliary strictures of the peripheral segmental bile duct may not require any treatment [126]. A bile leak could occur as a result of direct injury; the formation of biloma increases the risk of secondary infection and hepatic abscess. MRI with both MR cholangiopancreatography (MRCP) and study with hepatospecific contrast (EOB) is the imaging choice to localize bile leaks.

Treatment of lesions near to the gallbladder may increase gallbladder perforation or acute cholecystitis risk [126]. CT and/or Us allow assessment of cholecystitis [126].

Treatment of lesions localized within segments VII and VIII could damage the biliary duct and the pleura, resulting in a biliary-pleural fistula [126]. This can be assessed with contrast study CT or MRI with EOB.

Mechanical complications are due to thermal injury and include diaphragmatic injury, gallbladder perforation, colon perforation, stomach perforation and can occur when breaking the pleural, pneumothorax or haemothorax. Diaphragm injury appears as pleural effusion, diaphragmatic thickening and diaphragmatic hernia [126]. Gastric and colonic injury cause wall oedema or perforation [126].

Hepatic abscess occurs in 0.3–2% of treated patients about 7–10 day after the procedure [126]. The ablated target may be infected due to tissue necrosis. US is the first diagnostic tool employed, although a contrast study CT allows a proper characterization.

## 5. Radiologists: How We Should Report

In the emergency setting, an effective communication of imaging data to referring physicians is crucial for patient care [140,141,142,143,144,145,146,147,148,149]. Despite the technical developments in the radiological setting, the radiology report, representing the most important feature of communication with clinical partners, has not progressed significantly [140,141,142,143,144,145,146,147,148,149]. It remains poorly structured and its quality depends on the radiologist and their experience with a particular field. Traditionally, radiology reports are created as non-structured free-text (FRT) presentations in narrative language. However, inconsistencies with regard to content, style, and presentation can hamper information transfer and diminish the clarity of the reports, which can in turn adversely affect the extraction of the required key information by the referring physician. Therefore, FRT should be organized and re-orientated toward structured reports (SR) [150,151,152,153,154,155,156,157,158,159]. According to the European Society of Radiology (ESR) paper on SR in radiology, the three main goals for moving from FTR to SR are quality, datafication/quantification and accessibility [150,151,152,153,154,155,156,157,158,159]. With regard to quality, this is correlated to standardization. Using a structured template to report all pertinent items for a specific field, allows correlation of radiological data with other key clinical data, leading to personalized medicine (datafication and quantification). With regard to accessibility, since radiological reports may be considered as a source of data, these data may be evaluated to obtain new biomarkers that could be analyzed in appropriate clinical contexts helping to devise potential new application domains. In this scenario, by using a dedicated report template, the radiologist has a list of predefined relevant items for the case at hand, ensuring that no important data is missed. This should not only guarantee a consistently high quality of final report, but also aid in the management of patients [150,151,152,153,154,155,156,157,158,159].

With regard to complications of ablation treatment, the radiologist should report the technical success, treatment efficacy, and complications [59]. The complications should be assessed according to the well-defined classification systems (e.g., (a) the Common Terminology Criteria for Adverse Events standards, (b) the Clavien–Dindo classification, (c) the Society of Interventional Radiology classification, and (d) the Cardiovascular and Interventional Radiological Society of Europe Quality Assurance Document and Standards for Classification of Complications). Complications should be categorized consistently according to severity and time of occurrence. An accurate classification of a patient, considering complications, enables how the patient should be treated to be determined as soon as possible, and standardized language makes this possible. In addition, standardized reporting enables comparison of data between different clinical studies, with a view to obtaining more accurate evaluation of the efficacy and efficiency of a treatment, considering the clinical characteristics of the patient presented in the report. Therefore, a shared template between the various scientific societies is needed.

## 6. RFA, MWA and Other Treatments

Surgical resection remains the gold standard for treatment of primary or metastatic liver tumors. However, most patients are not candidates for hepatic resection because of anatomic limitations, the multifocal nature of the disease, insufficient functional liver reserve, extrahepatic metastases, or comorbidities. Surgical resection morbidity rates range from 20 to 56%, depending on the patient, the extent of resection, the disease process, the hospital and the surgeon [157,158,159,160,161,162,163,164,165,166,167]. As described by Benzoni et al. [161], during major hepatectomies, Pringle manoevres longer than 20 min and blood transfusion greater than 600 mL, were associated with significant increases in complications. Additionally, Childs B and C classification and histopathologic grading were associated with increased complications in patients with HCC [161]. Sadamori et al. [162] found significantly higher rates of bile leakage (12.8% overall) and organ/space surgical-site infections (8.6% overall) in patients undergoing repeat hepatectomy and prolonged surgery.

Considering these issues, RFA and MWA have become recognized treatment approaches because of their efficacy, reproducibility, low complication rates, and availability. The benefits of MWA are an improved convection profile, higher constant intratumoral temperatures, faster ablation times, and the ability to use multiple probes to treat multiple lesions simultaneously [24]. MWA target area size and shape may be more consistent and less dependent on the heat-sink effect from vascular structures in proximity of the lesion. RFA has been available for some time and is the more established thermal technique, but lesions with a diameter >2–2.5 cm need multiple overlapping ablations, and a subcapsular or high-risk location of a tumor is considered a relative contraindication to RFA [24]. MWA should be preferred when tumor size is ≥3 cm in diameter or in cases of lesions near to large vessels independently of size. Moreover, MWA can reach larger ablation volumes without a heat-sink effect [24].

In contrast to RFA and MWA, IRE and ECT [21,23,39,40] are non-thermal techniques that cause ablation by changing cell membrane permeability using an induced electric field (electroporation). IRE is considered a direct ablation tool, since electroporation is used in an irreversible manner, causing the irreversible permeabilization of the lipid bilayer, the disruption of cellular homeostasis and stimulation of apoptotic pathways, causing death of neoplastic cells. IRE can protect surrounding structures, such as the vessels, and is particularly valuable when the tumor encases major vessels [168,169,170]. ECT is based on cell electroporation combined with administration of a single dose of non-permeant or poorly permeant chemotherapeutic agents [39,40]. Electrical field application to a cell causes a transient and reversible orientation of its polar membrane molecules, with an increase in permeability. This transient increase in permeability allows the chemotherapeutic drugs to enter in the cell, thus increasing the cytotoxic effects of the agents. This local potentiation can increase chemotherapy efficacy [39,40]. Only a small number of studies are available on the efficacy and efficiency of these procedures in liver cancer; however, these could be considered alternative techniques in conditions such as proximity to the great vessels [171,172,173,174,175,176,177].

## 7. Conclusions

Ablation therapies for HCC and liver metastases have presented a challenge for radiologists, who need to assess the potential for complications related to treatment. To date, RFA and MWA have not been found to be statistically significantly different in terms of mortality or major or minor complication rates. To reduce major complication incidence, patient selection and risk assessment based on lesion evaluation are essential; patients at high-risk for infections, coagulation disorders, and previous abdominal surgery should be evaluated to establish the right cost-benefit ratio for the ablation method. Based on risk, lesion assessment with respect to tumor location in segments IVa or VIII, contact with the gallbladder, proximity of large blood and biliary vessels, proximity to extrahepatic structures, such as the pleura or/and intestine, as well as lesion size, is required in order to define suitable patient management, and to select alternative non-thermal treatment. During the procedure, with respect to surveillance, radiologists should pay attention to complications, such as vascular, biliary, mechanical and infectious complications. Multiphase CT is the imaging tool to choose in an emergency setting. The radiologist should report the technical success, treatment efficacy, and complications. The complications should be assessed according to well-defined classification systems, and these complications should be categorized consistently according to severity and time of occurrence.

## Figures and Tables

**Table 1:** RFA and MWA characteristics.

Treatment	RFA	MWA
Physical phenomenon to generate heat	Thermocoagulation necrosis	Dielectric heating
Necrosis volume	Restricted to areas of high current density; the zone of active tissue heating is limited to a few millimeters surrounding the active electrode, with the remainder of the ablation zone being heated via thermal conduction	Volume around the applicator antenna; up to 2 cm surrounding the antenna
Heat-sink effect	Yes	No
Benefits	Safety, tolerability, efficacy, ease of use, and cost-effectiveness	Similar benefits to RFA, with several advantages, such as a greater volume of cellular necrosis, procedure time reduction, and higher temperatures delivered to the target lesion, and reduced susceptibility to variation in the morphology of the treatment zone because of heat-sink effects from adjacent vasculature
Metastasis complication rates	Between 1.1% and 24%	Between 3.1% and 27%
HCC complication rates	Between 0% vs. 45.4%	Between 2.2% and 61.5%

## Data Availability

The data are available at link https://zenodo.org/record/6541403#.Yn38K-hBy3A.
